# Steam explosion-induced remodeling of volatile metabolites in *Lysimachia foenum-graecum* essential oil revealed by GC-MS and chemometric analysis

**DOI:** 10.3389/fpls.2026.1962102

**Published:** 2026-10-15

**Authors:** Lei Li, Yongzhen Zhao, Xiao Chen, Changtong Lu, Jiaming Zhao, Haoyang Chen, Gaolei Xi, Pengfei Yang, Miao Liang, Qingfu Wang, Yibo Ning

**Affiliations:** 1 China Tobacco Henan Industrial Co., Ltd., Zhengzhou, China; 2 College of Tobacco Science and Engineering, Zhengzhou University of Light Industry, Zhengzhou, China

**Keywords:** aroma profile, chemometric analysis, GC-MS, *Lysimachia foenum-graecum*, physicochemical stress, steam explosion, volatile metabolites

## Abstract

*Lysimachia foenum-graecum* is an aromatic plant rich in diverse volatile metabolites; however, the complexity of its volatile composition and chemical variability remain challenges for understanding and improving its aromatic characteristics. This study aimed to investigate steam explosion-induced remodeling of volatile metabolites in *L. foenum-graecum* essential oils from Guangxi and Yunnan origins. Steam explosion conditions were optimized using single-factor experiments combined with an orthogonal experimental design, and volatile profiles of essential oils before and after treatment were characterized by gas chromatography-mass spectrometry (GC-MS). Multivariate statistical analyses were further applied to identify differential volatile compounds associated with steam explosion treatment. Although the individual optima observed in the single-factor experiments differed from those of the final combination, orthogonal optimization identified a residence time of 60 s, a moisture addition ratio of 30%, and a steam pressure of 1.5 MPa as the optimal combination, yielding an essential oil yield of 0.603% ± 0.009%. A total of 95 volatile compounds were identified from essential oils of the two geographical origins. Although the total volatile content decreased after steam explosion treatment, distinct alterations in volatile metabolite profiles were observed, characterized by the selective reduction of phenolic compounds and long-chain fatty acids and their derivatives. OPLS-DA clearly differentiated untreated and treated samples, and 20 key differential compounds were identified using VIP > 1 and *p* < 0.05, most of which were long-chain fatty acids and their derivatives, including cis-9-hexadecenoic acid, methyl linoleate, methyl linolenate, and (E)-phytol. Despite the reduction in the total GC-MS-detectable volatile content, steam explosion altered the volatile composition of *L. foenum-graecum*. Sensory evaluation showed trends toward increased herbal and roasted attributes, although these changes were not statistically significant. This study provides new insights into steam explosion-induced regulation of volatile metabolite composition in aromatic plants and highlights its potential as a physicochemical strategy for modulating the chemical profiles of *L. foenum-graecum*.

## 1 Introduction

*Lysimachia foenum-graecum* Hance is an aromatic medicinal plant that has been traditionally used in spices, food flavoring, and natural product applications (Li et al., 2009; Qin et al., 2024). As a typical aromatic plant, *L. foenum-graecum* contains abundant volatile organic compounds, which represent an important class of plant specialized metabolites involved not only in aroma formation but also in ecological functions, including plant defense, environmental adaptation, and interactions with surrounding organisms (Abbas and Wang, 2025; Li et al., 2026; Wang et al., 2026). Its characteristic odor is mainly attributed to a diverse group of volatile compounds, including terpenoids, fatty acid derivatives, and phenolic substances (Dai et al., 2023). The composition and relative abundance of these compounds determine the overall aroma profile and sensory properties of *L. foenum-graecum*-derived products. However, the accumulation and distribution of plant volatile metabolites are highly dynamic and can be influenced by genetic background, environmental conditions, and processing-induced stresses (Al-Amri, 2026; Escobar-Bravo et al., 2023). Plant-derived volatile mixtures are usually highly complex, and interactions among different compounds may result in unstable aroma characteristics and insufficient flavor identity during processing (Abbas et al., 2023; Chiaia et al., 2025). Therefore, investigating the variation of volatile metabolites in *L. foenum-graecum* is essential for understanding the chemical diversity of aromatic plants and their responses to physicochemical stresses.

Various processing strategies, such as extraction optimization, thermal treatment, enzymatic modification, and fermentation, have been explored to regulate aroma-related compounds in plant materials (Fang et al., 2026; Huang et al., 2026; Lou et al., 2025; Saeed et al., 2026). Although these approaches can alter volatile profiles to some extent, limitations remain regarding processing efficiency, selective regulation of target compounds, and the possible loss of heat-sensitive constituents. In recent years, physical processing technologies capable of inducing structural and chemical modifications in plant tissues have attracted increasing attention as potential approaches for regulating plant specialized metabolites (Chen et al., 2026). Steam explosion, an emerging physicochemical pretreatment technology, has recently received considerable attention in plant processing (Wan et al., 2022; Wang et al., 2026). This process involves exposing plant tissues to high-pressure steam followed by rapid depressurization, which induces mechanical disruption and structural modification of plant matrices, thereby enhancing the accessibility and release of intracellular metabolites (Li et al., 2023). Beyond these physical effects, the combination of elevated temperature, high pressure, saturated steam, and rapid decompression imposes a strong abiotic physicochemical stress on plant tissues, which may induce chemical transformations and perturbations in secondary metabolism (Su et al., 2025; Yu and Jia, 2025).

Abiotic stresses, including heat, drought, salinity, and mechanical injury, are known to alter plant volatile emissions through changes in terpenoid and lipid-derived metabolic processes (Loreto and Schnitzler, 2010). In this context, steam explosion may similarly perturb volatile metabolism, although its effects differ from conventional environmental stresses in terms of intensity and timescale. The resulting changes in volatile composition may arise from multiple concurrent processes, including the degradation or loss of thermally labile volatiles, the release of bound aroma precursors, and the formation or transformation of volatile compounds. These processes may involve pathways associated with volatile secondary metabolism, such as the lipoxygenase (LOX), methylerythritol phosphate (MEP), and mevalonate (MVA) pathways (Shan and Jin, 2025; Copolovici et al., 2012). Therefore, the volatile profile following steam explosion likely reflects a combined outcome of physicochemical transformation, precursor release, and stress-associated metabolic perturbation rather than simple enhancement of volatile extraction (Wang et al., 2026; Xue et al., 2024; Ye et al., 2026; Yu and Jia, 2025). However, the extent to which steam explosion-induced physicochemical stress contributes to changes in plant volatile metabolism remains insufficiently understood. Comprehensive characterization of volatile compounds is essential for elucidating processing-induced changes in aroma composition and quality. Combined with chemometric approaches, such as orthogonal partial least squares-discriminant analysis (OPLS-DA) and variable importance in projection (VIP), volatile profiling provides an effective strategy for identifying key compounds responsible for aroma differentiation and revealing the complex interactions among aroma-related components (Liang et al., 2025). Previous studies of steam explosion have primarily focused on lignocellulosic deconstruction, biomass conversion, enhanced extraction efficiency, and the release of phenolic or other bioactive constituents (Golmohammadi et al., 2018; Yu et al., 2020). In contrast, considerably less is known about how this high-temperature/high-pressure treatment reshapes the complex volatile composition of aromatic plant materials. In particular, whether different classes of volatile and semi-volatile constituents undergo selective loss, release, or transformation during steam explosion, and whether materials from different geographical origins respond differently under identical processing conditions, remain insufficiently characterized (Fu et al., 2026; Ye et al., 2026). Addressing these questions is important for understanding processing-induced chemical remodeling of aromatic plant resources and for evaluating steam explosion as a tool for directional aroma modulation (Wang et al., 2026).

Accordingly, this study aims to investigate the potential of steam explosion pretreatment for regulating the volatile profile and aroma characteristics of *L. foenum-graecum* essential oil. The specific objectives were to: (1) optimize the steam explosion pretreatment conditions for *L. foenum-graecum* based on essential oil yield using single-factor experiments combined with an orthogonal experimental design; (2) characterize steam explosion-induced changes in volatile metabolite profiles of essential oils from Guangxi and Yunnan *L. foenum-graecum* using GC-MS analysis; and (3) identify key differential volatile compounds associated with steam explosion-induced metabolic remodeling through multivariate statistical analyses. From an application perspective, establishing the response of *L. foenum-graecum* aroma constituents to steam explosion may support the development of controlled pretreatment strategies for spice and aromatic plant processing. Such information may facilitate directional modulation of aroma characteristics while allowing processing conditions to be selected by balancing desirable sensory changes against losses of volatile constituents.

## 2 Materials and methods

### 2.1 Materials

*L. foenum-graecum* leaves were harvested at the mature stage from Guilin (Guangxi) and Wenshan (Yunnan), China, in September 2025. After collection, the samples were sun-dried and subsequently stored at 4 °C prior to analysis. Ultrapure water was obtained from Wahaha Group Co., Ltd. A C7–C40 n-alkane standard mixture (99.6% purity) purchased from Beijing J&K Scientific Ltd. was used for the calculation of retention indices (RIs) of volatile compounds. Anhydrous sodium sulfate (analytical grade) obtained from Yantai Shuangshuang Chemical Co., Ltd. was used for dehydration of volatile extracts.

Dichloromethane and absolute ethanol were purchased from Sinopharm Chemical Reagent Co., Ltd. 2,6-Dichlorotoluene (Beijing J&K Scientific Ltd.) was used as the internal standard for volatile compound quantification. Authentic standards used for volatile compound identification were obtained from Achem-block, Larodan, Sigma-Aldrich, and Adamas. The purity of these standards ranged from 92% to 99.9%.

### 2.2 Methods

#### 2.2.1 Steam explosion pretreatment of *L. foenum-graecum*

The *L. foenum-graecum* samples were cut into approximately 5cm segments. A 100 g portion of sample was accurately weighed, and the moisture content was adjusted by adding an appropriate amount of distilled water according to the experimental design. The samples were then subjected to steam explosion pretreatment using a laboratory-scale steam explosion reactor (QBS-80, Suzhou Qingzheng Ecological Technology Co., Ltd., Suzhou, China) (**Supplementary Figure S1**). The main processing parameters, including steam pressure, residence time, and moisture content, were determined based on single-factor experiments and orthogonal experimental design. After treatment, the steam-exploded samples were rapidly cooled and stored at 4 °C until further analysis.

#### 2.2.2 Essential oil preparation from untreated and steam-exploded *L. foenum-graecum*

The untreated and steam-exploded samples were air-dried and ground into powder using a grinder. The powder was passed through a 120-mesh sieve before essential oil extraction. Essential oils were extracted by hydrodistillation. Briefly, 10 g of sample powder was placed into a round-bottom flask, and distilled water was added at a solid-to-liquid ratio of 1:20 (g/mL). The mixture was subjected to hydrodistillation for 20 h after the onset of boiling. The obtained crude essential oil was extracted with dichloromethane, followed by dehydration with anhydrous sodium sulfate overnight. The solvent was subsequently removed by evaporation under atmospheric pressure to obtain the purified essential oil.

The essential oil yield was calculated as follows:


$$\text{Essential oil yield }(\%)=\frac{\text{Weight of extracted essential oil}}{\text{Weight of dry sample}}\times 100$$

Three independent steam explosion experiments were conducted under the optimized conditions, and the resulting samples were analyzed by GC-MS, and the mean values were used for subsequent analysis.

#### 2.2.3 Single-factor optimization of steam explosion parameters

Single-factor experiments were conducted to evaluate the effects of individual steam explosion parameters on the essential oil yield of *L. foenum-graecum*. Briefly, 100 g of samples were accurately weighed and subjected to steam explosion treatment. The effects of three processing variables, including moisture content (15%, 30%, 45%, and 60%), residence time (30, 60, 90, and 120 s), and steam explosion pressure (0.5, 1.0, 1.5, and 2.0 MPa), were investigated while maintaining other parameters constant. The essential oils obtained from treated samples were extracted according to the procedure described in Section 2.2.2. The essential oil yield was selected as the evaluation index to determine the suitable ranges of steam explosion parameters for subsequent optimization.

#### 2.2.4 Optimization of steam explosion conditions using orthogonal experimental design

Based on the results of single-factor experiments, an orthogonal experimental design L16(4^5^) was employed to optimize the steam explosion pretreatment conditions of *L. foenum-graecum*. The essential oil yield obtained after steam explosion treatment was used as the response variable. Residence time, moisture addition ratio, and steam pressure were selected as the major processing variables because they jointly determine heat transfer, steam penetration, internal vaporization, and decompression intensity during steam explosion. The four levels of each factor were established based on preliminary trials and reported steam-explosion processing ranges, thereby covering conditions from relatively mild to severe treatment while maintaining operational feasibility. The detailed experimental design is presented in **Table 1**. The predicted optimal combination was subsequently validated using three independent steam-explosion and extraction experiments, and the essential oil yield is reported as mean ± SD (n = 3).

**Table 1:** Factors and levels of the orthogonal experimental design for steam explosion pretreatment of *Lysimachia foenum-graecum*.

Level	A: Residence time (s)	B: Moisture addition ratio (%)	C: Steam explosion pressure (MPa)
1	30	15	0.5
2	60	30	1.0
3	90	45	1.5
4	120	60	2.0

#### 2.2.5 GC-MS analysis and identification of volatile compounds

The volatile compounds in *L. foenum-graecum* essential oils before and after steam explosion treatment were analyzed using GC-MS.

GC conditions: Volatile compounds were separated using an Agilent DB-5MS capillary column (60 m × 250 μm × 0.25 μm). The injector temperature was maintained at 280 °C. High-purity helium (99.999%) was used as the carrier gas at a constant flow rate of 1.0 mL/min. Samples were injected in split mode with a split ratio of 10:1, and the injection volume was 1 μL. The oven temperature program was as follows: the initial temperature was set at 40 °C, followed by an increase to 140 °C at a rate of 1.5 °C/min and held for 15 min. The temperature was then increased to 200 °C at 3 °C/min and maintained for 10 min, followed by a final increase to 280 °C at 3 °C/min. The relatively low initial oven temperature and slow heating rate in the first stage were selected to improve separation of relatively volatile constituents, whereas the subsequent increase to 280 °C enabled elution of less volatile, higher-molecular-weight constituents such as long-chain fatty acids and their derivatives. Nevertheless, highly volatile compounds may have been partially affected by sample preparation and solvent-removal procedures before GC-MS analysis.

MS conditions: Mass spectrometric analysis was performed under electron ionization (EI) mode at an electron energy of 70 eV. The ion source temperature, quadrupole temperature, and transfer line temperature were maintained at 230, 150, and 280 °C, respectively. Data acquisition was performed in full scan mode with a mass range of m/z 35–700. The solvent delay time was set at 8 min.

Identification of volatile compounds: The volatile compounds were tentatively identified by comparing the mass spectra obtained from the total ion chromatograms (TICs) with the NIST 20 mass spectral library. Compounds with a matching factor above 75 were initially selected and further confirmed by comparing their retention times and RIs with those of authentic standards.

Quantification of volatile compounds: Relative quantification of volatile compounds was performed using the internal standard method (2,6-dichlorotoluene, 0.65 mg/mL). The relative concentration of each volatile compound was calculated based on the peak area ratio between the target compound and internal standard according to the following equation:


$$w=\frac{S_{0}\times W_{1}}{S_{1}}$$

where: w represents the concentration of volatile compounds (μg/g); $S_{0}$represents the peak area of the target compound; $S_{1}$represents the peak area of the internal standard; $W_{1}$represents the concentration of internal standard (μg/g).

#### 2.2.6 Sensory evaluation of essential oils

Sensory evaluation was performed according to GB/T 14454.2–2008 with minor modifications. The aroma characteristics of *L. foenum-graecum* essential oils obtained under different treatments were evaluated. The panel consisted of 11 participants (six males and five females) who were screened for normal olfactory function, availability, and ability to discriminate the selected aroma attributes. Before formal evaluation, the panelists participated in three training sessions of approximately 45 min each. During training, reference materials representative of the selected descriptors was introduced, and panelists practiced descriptor recognition and intensity scoring until the coefficient of variation (CV) among panelists for each attribute fell below 20%, indicating acceptable agreement. The three training sessions and the use of averaged scores from 11 panelists were considered sufficient to ensure panel consistency for the comparative sensory profiling in this study. The initial descriptor pool was established based on preliminary sniffing of *L. foenum-graecum* essential oils. Following panel discussion and removal of redundant descriptors, six attributes were retained for formal evaluation: herbal, floral, sweet, balsamic, woody, and roasted. The evaluation was conducted in a sensory laboratory under white illumination at 18 °C. Samples were randomly coded and presented to panelists in a blind manner. Aroma intensity was scored using a nine-point scale ranging from 0 (not detectable) to 9 (extremely strong). Each essential oil sample was evaluated by all 11 panelists in a single session per treatment, with each panelist providing one intensity score per attribute per sample. The final aroma intensity score for each attribute was calculated as the mean of the 11 individual scores.

#### 2.2.7 Statistical analysis

Statistical analyses were performed using SPSS 27.0 (IBM, Armonk, NY, USA). Data are expressed as mean ± standard deviation based on three independent experiments unless otherwise stated. For each compound, differences among the four sample groups were evaluated using one-way ANOVA followed by Tukey’s HSD post-hoc test. A *p* value < 0.05 was considered statistically significant. OPLS-DA and VIP analyses were conducted on the Pareto-scaled semi-quantitative concentration matrix of the detected compounds using SIMCA 14.1 (Umetrics, Umeå, Sweden). A 200-permutation test was used to evaluate the robustness of the OPLS-DA model. Differential compounds were screened using VIP > 1 combined with *p* < 0.05. Hierarchical cluster analysis (HCA) was performed using TBtools. Euclidean distance was used as the distance metric, and the between-groups linkage method was applied for cluster aggregation. Both samples and volatile compounds were clustered, and the data were standardized prior to analysis. Origin 2021 (OriginLab, Northampton, MA, USA) were used to create line charts, bar graphs, radar charts.

## 3 Results and discussion

### 3.1 Optimization of steam explosion conditions for *L. foenum-graecum* pretreatment

#### 3.1.1 Effect of steam explosion pressure on essential oil yield

As shown in **Figure 1a**, steam explosion pressure affected the essential oil yield of *L. foenum-graecum* under a fixed residence time of 60 s and moisture addition ratio of 30%. Among the investigated pressure levels, the highest essential oil yield was obtained at 1.0 MPa, whereas both lower (0.5 MPa) and higher pressures (1.5 and 2.0 MPa) resulted in relatively lower yields. These results indicate that an appropriate steam explosion pressure is critical for enhancing essential oil release (Golmohammadi et al., 2018; Zhang et al., 2019). The increased yield at 1.0 MPa may be attributed to the moderate mechanical disruption caused by steam explosion, which promotes cell wall rupture and facilitates the release of essential oils (Ramadhan et al., 2026). However, excessive pressure may induce stronger thermal and mechanical stress, leading to the degradation or loss of volatile compounds and consequently reducing the extractable essential oil yield (Kristiawan et al., 2008).

**Figure 1:**
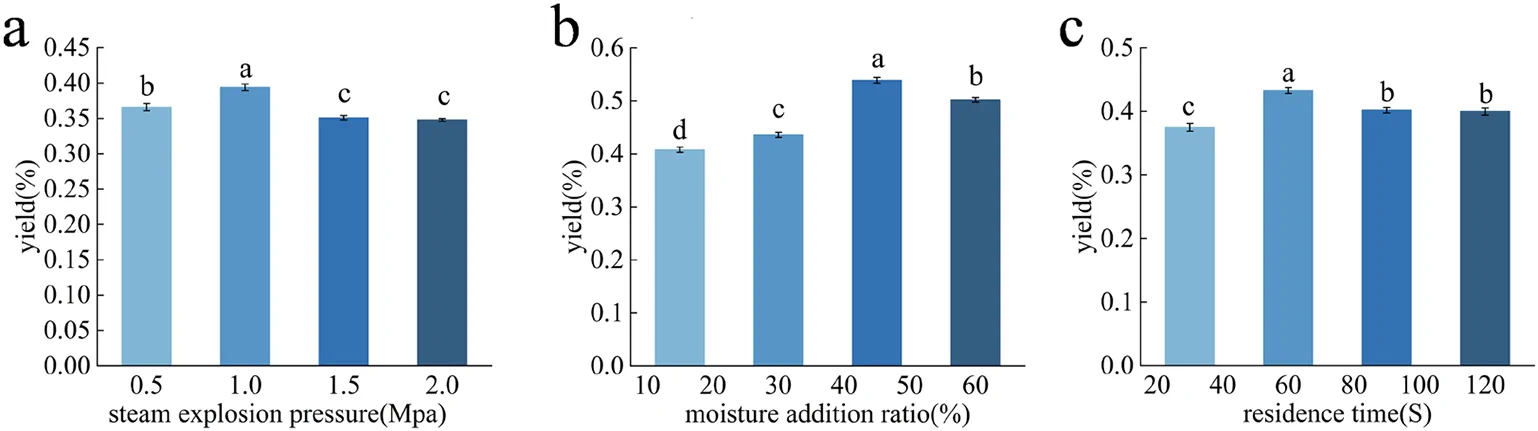
Effects of **(a)** steam explosion pressure, **(b)** moisture addition ratio, and **(c)** residence time on the extraction yield of *Lysimachia foenum-graecum* essential oil.

#### 3.1.2 Effect of moisture addition ratio on essential oil yield

The effect of moisture addition ratio on essential oil yield is shown in **Figure 1b**. Under a fixed residence time of 60 s and steam explosion pressure of 1.0 MPa, the essential oil yield varied significantly among different moisture addition ratios. The highest yield was obtained at a moisture addition ratio of 45%, while both lower (15% and 30%) and higher moisture levels (60%) resulted in relatively lower yields. These results suggest that an appropriate moisture level is essential for achieving efficient steam explosion pretreatment. The improvement in essential oil yield at 45% moisture addition may be attributed to enhanced heat transfer and steam diffusion within plant tissues (Sui and Chen, 2015). During the sudden depressurization process, sufficient internal moisture can rapidly vaporize and generate an expansion force, facilitating cell structure disruption and promoting the release of essential oils (Sui and Chen, 2016). However, excessive moisture addition may reduce the effective pressure difference during steam explosion and dilute the mechanical disruption effect, thereby limiting the release of volatile compounds (Liu et al., 2017).

#### 3.1.3 Effect of residence time on essential oil yield

As shown in **Figure 1c**, the highest essential oil yield was obtained at 60 s, whereas shorter (30 s) and longer times (90 and 120 s) resulted in relatively lower yields. These results indicate that an appropriate residence time is required to achieve efficient release of essential oils during steam explosion pretreatment. The enhanced yield at 60 s may be attributed to sufficient heat transfer between steam and plant tissues, which promotes cell wall disruption and facilitates the migration of essential oils (Chen et al., 2012). However, excessive residence time may lead to prolonged exposure of volatile compounds to high-temperature steam, accelerating thermal degradation and volatilization losses, thereby reducing the extractable essential oil yield (Jacquet et al., 2011).

### 3.2 Optimization of steam explosion conditions using orthogonal experimental design

Based on the results of single-factor experiments, residence time (A), moisture addition ratio (B), and steam explosion pressure (C) were selected as the main variables for further optimization. An L16(4^5^) orthogonal experimental design was conducted using essential oil yield as the response value. The results and range analysis are presented in **Table 2**.

**Table 2:** Orthogonal experimental design and range analysis for optimization of steam explosion pretreatment of *Lysimachia foenum-graecum*.

Nos.	Factors					Yield/%
	A	B	C	D (Blank)	E (Blank)	
1	1 (30)	1 (15)	1 (0.5)	1	1	0.269
2	1 (30)	2 (30)	2 (1.0)	2	2	0.352
3	1 (30)	3 (45)	3 (1.5)	3	3	0.394
4	1 (30)	4 (60)	4 (2.0)	4	4	0.365
5	2 (60)	1 (15)	2 (1.0)	3	4	0.416
6	2 (60)	2 (30)	1 (0.5)	4	3	0.536
7	2 (60)	3 (45)	4 (2.0)	1	2	0.439
8	2 (60)	4 (60)	3 (1.5)	2	1	0.469
9	3 (90)	1 (15)	3 (1.5)	4	2	0.39
10	3 (90)	2 (30)	4 (2.0)	3	1	0.458
11	3 (90)	3 (45)	1 (0.5)	2	4	0.364
12	3 (90)	4 (60)	2 (1.0)	1	3	0.293
13	4 (120)	1 (15)	4 (2.0)	2	3	0.408
14	4 (120)	2 (30)	3 (1.5)	1	4	0.566
15	4 (120)	3 (45)	2 (1.0)	4	1	0.317
16	4 (120)	4 (60)	1 (0.5)	3	2	0.374
K_1_	1.38	1.483	1.543	1.567	1.513	
K_2_	1.86	1.912	1.378	1.593	1.555	
K_3_	1.505	1.514	1.819	1.642	1.631	
K_4_	1.665	1.501	1.67	1.608	1.711	
k_1_	0.345	0.37075	0.38575	0.39175	0.37825	
k_2_	0.465	0.478	0.3445	0.39825	0.38875	
k_3_	0.37625	0.3785	0.45475	0.4105	0.40775	
k_4_	0.41625	0.37525	0.4175	0.402	0.42775	
R	0.12	0.10725	0.11025	0.01875	0.0495	
Priority order	A>C>B					
Optimal level	A2	B2	C3			
Optimal combination	A2B2C3					

*Blank columns D and E in orthogonal array are reserved for error estimation.

As shown in **Table 2**, the essential oil yield varied considerably among different experimental combinations, ranging from 0.269% to 0.566%. The range analysis indicated that the range values of the three investigated factors were higher than those of the empty columns, suggesting that all selected parameters contributed to variations in essential oil yield. Among the three factors, residence time exhibited the greatest influence (R = 0.120), followed by steam explosion pressure (R = 0.11025) and moisture addition ratio (R = 0.10725). Therefore, the order of influence was determined as:

Residence time > Steam explosion pressure > Moisture addition ratio

Based on the average response values at different levels, the optimal combination was determined as A2B2C3, corresponding to a residence time of 60 s, moisture addition ratio of 30%, and steam explosion pressure of 1.5 MPa. Three replicate validation experiments were subsequently performed under the optimized conditions, resulting in an essential oil yield of 0.603% ± 0.009%. Since A_2_B_2_C_3_ was not among the 16 tested runs, this higher validation yield (compared with the array maximum of 0.566%) is consistent with the purpose of orthogonal optimization, and the low variation across replicates further confirmed the reproducibility of this condition. The optimal levels obtained from single-factor experiments did not completely coincide with the final orthogonal combination. This discrepancy is expected, as single-factor experiments vary one factor with others held constant, whereas orthogonal designs assess factor levels across variable combinations. Thus, the superior performance of 1.5 MPa in the A_2_B_2_C_3_ combination reflects a multifactorial outcome within the tested space, rather than an inherent advantage of 1.5 MPa over 1.0 MPa under all conditions. Consistent with previous steam explosion optimization studies, all investigated factors significantly affected the essential oil yield in the present work, and the validation yield exceeding the array maximum confirmed the effectiveness of orthogonal optimization. Similar to orange peel essential oil extraction, where both pressure and temperature were influential (Golmohammadi et al., 2018), residence time, pressure, and moisture addition ratio all contributed significantly to essential oil yield in this study. Bamboo fiber optimization likewise showed that multiple factors can simultaneously affect the response (*p* < 0.01), further supporting the substrate-dependent nature of steam explosion performance (Zhao et al., 2024). Notably, moisture addition ratio was included as an independent factor, distinguishing this work from previous essential oil studies in which moisture was fixed or not separately evaluated.

As the L16(4^5^) design did not independently resolve A×B, A×C, or B×C interactions, quantitative interpretation of pairwise interactions is not feasible in the present study. Future optimization may therefore benefit from response-surface or full factorial designs to further explore factor interactions. To further evaluate the statistical significance of each processing factor, analysis of variance (ANOVA) was performed, and the results are shown in **Table 3**. Residence time (A), moisture addition ratio (B), and steam explosion pressure (C) significantly affected essential oil yield. Specifically, residence time and moisture addition ratio showed highly significant effects (*p* < 0.01), whereas steam explosion pressure exhibited a significant effect (*p* < 0.05). These results indicate that the duration of steam treatment, water availability, and pressure conditions collectively determine the extent of structural disruption and volatile compound release during steam explosion pretreatment.

**Table 3:** Analysis of variance (ANOVA) for the effects of steam explosion parameters on essential oil yield.

Factors	SS	df	MS	F-value	*p*-value	Significance^a^
A	0.032	3	0.011	10.021	0.009	**
B	0.032	3	0.011	9.9411	0.009	**
C	0.026	3	0.009	8.171	0.015	*
Error	0.006	6	0.001			

a. **means *p* < 0.01, *means *p* < 0.05.

### 3.3 Changes in volatile metabolite profiles of *L. foenum-graecum* essential oils induced by steam explosion

Based on the optimized steam explosion conditions obtained from single-factor and orthogonal experiments, samples from Guangxi and Yunnan were subjected to steam explosion pretreatment. Essential oils were subsequently extracted according to the procedure described in Section 2.2.2. The essential oils obtained before and after treatment were designated as GSEB and GSEA for Guangxi samples, and YSEB and YSEA for Yunnan samples, respectively. The volatile profiles were analyzed using GC-MS, and the results are presented in **Supplementary Table S1**, **Table 4**. Because dichloromethane was removed by evaporation after extraction, partial loss of highly volatile, low-boiling constituents cannot be excluded. Therefore, the GC-MS profiles obtained in this study primarily represent constituents retained under the standardized extraction and concentration conditions rather than exhaustive recovery of the complete native volatile. A total of 95 volatile compounds were identified in the four essential oil samples. Among them, 72 compounds were detected in both Guangxi and Yunnan samples, although differences in composition and relative abundance were observed between the two origins, indicating that geographical origin may contribute to variations in volatile profiles. Comparison of samples before and after steam explosion revealed that the total volatile contents decreased after treatment. Specifically, the total volatile content of Guangxi essential oil decreased from 18,655.70 μg/g to 13,784.12 μg/g, corresponding to a reduction of 26.1%. Similarly, the volatile content of Yunnan essential oil decreased from 21,034.37 μg/g to 16,311.82 μg/g, with a reduction of 22.5%. According to the total ion chromatograms (**Figure 2**), most compounds showing decreased abundance after steam explosion exhibited retention times below 70 min. These compounds, mainly including linalool, borneol, α-terpineol, decanal, undecanal, and geranylacetone, were eluted before the oven temperature reached approximately 140 °C according to the programmed temperature conditions, suggesting that highly volatile compounds may undergo evaporation loss or thermal degradation during steam explosion treatment. The altered chromatographic profile after steam explosion likely represents the combined outcome of several processes rather than simple thermal degradation. First, relatively volatile or thermolabile constituents may be lost through evaporation or degradation during high-temperature treatment and subsequent sample preparation. Second, disruption of cell walls, membranes, and intracellular structures may facilitate the release of compounds or bound precursors that were less extractable in untreated material. Third, thermal and pressure-associated reactions may generate new chemical species through precursor conversion. The present GC-MS data cannot quantitatively distinguish the contribution of these processes; therefore, these mechanisms should be regarded as plausible explanations for the observed compositional remodeling (Mahanta et al., 2021; Szliszka et al., 2009).

**Table 4:** Primary volatile compounds detected in *Lysimachia foenum-graecum*.

CAS	Compounds	GSEB (ug/g)	GSEA (ug/g)	YSEB (ug/g)	YSEA (ug/g)	VIP	Significance^b^
78-70-6	Linalool	425.99 ± 9.43	336.79 ± 12.96	681.75 ± 7.86	397.44 ± 5.63	1.35	***
502-69-2	Perhydrofarnesyl acetone	459.93 ± 3.64	60.09 ± 1.79	1093.86 ± 34.86	981.42 ± 24.67	2.12	***
505-32-8	Isophytol	287.69 ± 4.67	162.82 ± 4.79	157.75 ± 1.47	458.72 ± 4.65	1.53	***
150-86-7	(E)-Phytol	1633.46 ± 14.36	1169.51 ± 19.65	1260.55 ± 41.86	1322.03 ± 24.68	1.64	***
10236-16-5	phytyl acetate	425.5 ± 11.54	370.3 ± 7.99	74.23 ± 4.53	78.08 ± 0.77	1.3	***
143-07-7	Lauric acid	141.08 ± 1.96	40.95 ± 0.98	276.81 ± 4.51	304.3 ± 4.86	1.09	***
373-49-9	cis-9-Hexadecenoic acid	223.52 ± 2.46	928.89 ± 8.43	150.31 ± 4.05	119.43 ± 4.33	2.11	***
57-10-3	Palmitic acid	4250.98 ± 16.77	3431.62 ± 17.66	6213.07 ± 143.78	4907.32 ± 50.63	3.41	***
506-12-7	Margaric acid	2402.75 ± 17.54	3132.04 ± 33.55	4370.91 ± 43.86	4351.61 ± 64.86	3	***
112-80-1	Pamolyn	915.71 ± 2.78	ND ^a^	ND	ND	2.3	***
60-33-3	Linoleic acid	1862.96 ± 24.65	184.46 ± 1.23	1525.37 ± 24.76	347.83 ± 5.65	3.02	***
463-40-1	Linolenic acid	2020.12 ± 36.87	1299.19 ± 40.76	1384.38 ± 33.88	15.85 ± 0.17	3.01	***
506-17-2	(Z)-11-Octadecenoic acid	ND	ND	ND	772.92 ± 45.69	2.27	***
112-44-7	Hendecanal	89.36 ± 3.11	68.95 ± 3.91	352.35 ± 5.78	2.66 ± 0.08	1.48	***
14010-23-2	Ethyl heptadecanoate	268.55 ± 2.67	18.82 ± 0.24	141.05 ± 7.43	53.59 ± 3.67	1.11	***
112-63-0	Methyl linoleate	100.16 ± 3.67	336.65 ± 6.95	106.45 ± 0.99	21.06 ± 0.57	1.31	***
301-00-8	Methyl linolenate	165.01 ± 1.58	584.98 ± 4.37	113.57 ± 3.46	ND	1.74	***
544-35-4	Ethyl linoleate	460.25 ± 5.66	222.63 ± 8.69	144.19 ± 1.86	42.76 ± 0.63	1.31	***
1191-41-9	Ethyl linolenate	543.83 ± 13.56	98.34 ± 0.76	143.03 ± 3.8	ND	1.58	***
1731-92-6	Methyl margarate	ND	121.76 ± 4.59	123.3 ± 4.54	29.68 ± 0.66	1.03	***

a. not detected.

b. ***means *p* < 0.001, **means *p* < 0.01, *means *p* < 0.05.

**Figure 2:**
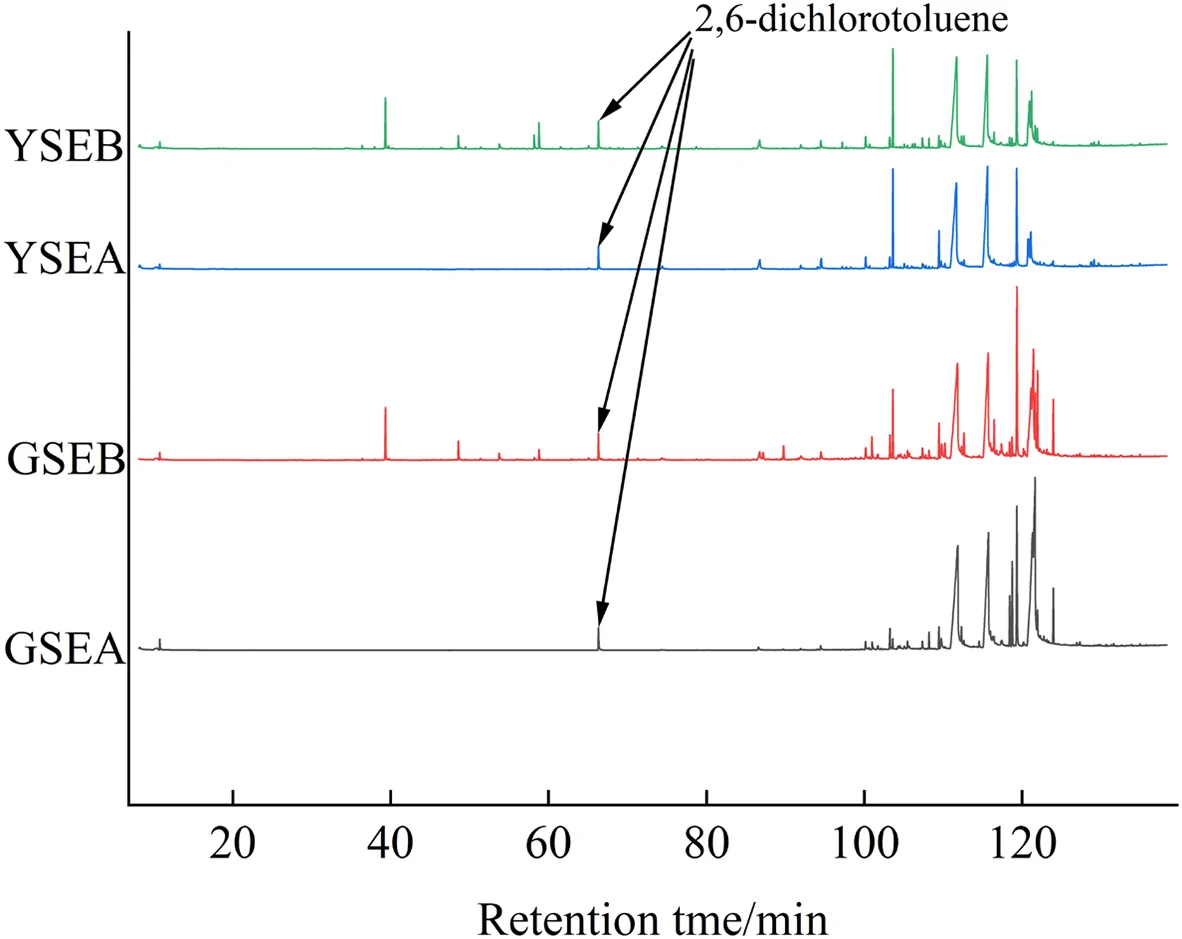
Total ion chromatograms of *Lysimachia foenum-graecum* essential oils before and after steam explosion.

#### 3.3.1 Terpenoids

Steam explosion induced distinct changes in terpenoid compounds of *L. foenum-graecum* essential oils depending on geographical origin. In Guangxi samples, the total terpenoid content decreased from 3981.42 to 2687.06 μg/g after treatment, corresponding to a reduction of 32.5%, whereas only an 8.2% decrease was observed in Yunnan samples (from 4109.72 to 3773.26 μg/g) (**Supplementary Table S1**). The distinct terpenoid responses between the two origins suggest that steam explosion efficacy depends on initial material characteristics. Genotype, tissue structure, cell-wall composition, moisture distribution, and precursor pools may differ across geographical populations and influence steam penetration, release efficiency, and terpenoid susceptibility to thermal conversion (Yang and Cadwallader, 2025). These variables were not independently quantified; thus, their contributions remain hypotheses requiring further investigation. At the individual compound level, perhydrofarnesyl acetone showed a substantial decrease in Guangxi samples (86.9% reduction), while its content remained relatively stable in Yunnan samples. In addition, the increased abundance of Isophytol in Yunnan samples may be associated with the release of volatile precursors or redistribution of metabolite components during steam explosion (Jiang et al., 2022). These differences may result from variations in tissue structure and metabolite microenvironments among different origins, suggesting that steam explosion induces selective remodeling of terpenoid volatile metabolites in *L. foenum-graecum*.

#### 3.3.2 Fatty acids

Fatty acid-related volatile metabolites showed an overall decreasing trend after steam explosion treatment. The total fatty acid content decreased from 12550.63 to 9364.25 μg/g in Guangxi samples and from 14843.89 to 11779.12 μg/g in Yunnan samples, corresponding to reductions of 25.4% and 20.7%, respectively (**Supplementary Table S1**). In addition, several long-chain fatty acids, including palmitic acid, linoleic acid, linolenic acid, and oleic acid, exhibited decreased levels after steam explosion (Yu et al., 2020). These compounds generally possess low volatility and weak aroma contribution, and their reduction may minimize interference from fatty-like odors, allowing characteristic aroma compounds to become more prominent (Chen and Chen, 2010). Saturated fatty acids remained relatively stable after treatment, whereas unsaturated fatty acids exhibited greater degradation. For example, linoleic acid in Guangxi samples decreased by 90.1%, and linolenic acid in Yunnan samples decreased by 98.9%. Interestingly, cis-9-hexadecenoic acid increased in the Guangxi samples after treatment. One possible explanation is that tissue disruption enhanced the extractability of lipid-associated constituents or that thermal processing altered the relative distribution of fatty-acid-related compounds. However, because lipid precursor pools and reaction pathways were not directly monitored, the mechanism underlying this increase cannot be established from the present data (Cheng et al., 2015; Zhang et al., 2019). Overall, steam explosion exerted a stronger effect on unsaturated fatty acids and induced selective remodeling of fatty acid-related volatile metabolites in *L. foenum-graecum*.

#### 3.3.3 Phenols

Phenolic compounds were detected only in Guangxi samples, whereas none were identified in Yunnan samples, indicating distinct phenolic profiles among different origins. After steam explosion treatment, the total phenolic content in Guangxi samples decreased from 61.24 to 14.04 μg/g, corresponding to a reduction of 77.1% (**Supplementary Table S1**), suggesting that these compounds were highly sensitive to high-temperature and high-pressure treatment. The absence of phenolic constituents in the Yunnan essential oil under the present conditions indicates an origin-associated difference. This variation could reflect differences in genetic background, growing environment, harvest stage, or postharvest handling, all of which affect metabolite accumulation. However, as these factors were not independently controlled, geographical origin should be regarded as an integrated source of variation rather than a single causal factor.

Among individual compounds, 4-ethylguaiacol, 2,6-dimethoxy-4-ethylphenol, and syringylpropane remained at low levels after treatment, whereas several phenolic compounds, including 4-ethylresorcinol, 3-methyl-5-ethylphenol, and carvacrol, were no longer detected. Since some phenolic compounds may contribute to pungent or undesirable aroma characteristics, their substantial reduction may contribute to the improvement of the overall aroma profile of *L. foenum-graecum* essential oil (Wang et al., 2026). Overall, steam explosion selectively regulated phenolic volatile compounds and further influenced the aroma composition of *L. foenum-graecum* from different origins (Miraballes et al., 2013; Wan et al., 2022).

### 3.4 Chemometric identification of steam explosion-induced volatile metabolite differences

To investigate the influence of steam explosion on the volatile composition of *L. foenum-graecum* essential oils, hierarchical clustering analysis (HCA) was performed using 95 identified volatile compounds as variables. As shown in **Figure 3**, the four samples were clearly separated into four clusters, indicating that both geographical origin and steam explosion treatment contributed to variations in volatile profiles. To further evaluate the differences among samples, OPLS-DA was conducted based on the 95 volatile compounds. As shown in **Figure 4a**, GSEB, GSEA, YSEB, and YSEA samples were clearly separated into four distinct regions, demonstrating that steam explosion significantly altered the volatile composition of *L. foenum-graecum* essential oils. This result echoes the 100% correct classification reported for geographical origin discrimination of Moroccan *Eucalyptus* essential oils using O2PLS-DA and HCA, further confirming the applicability of this analytical framework to volatile profiling studies (El Orche et al., 2024).

**Figure 3:**
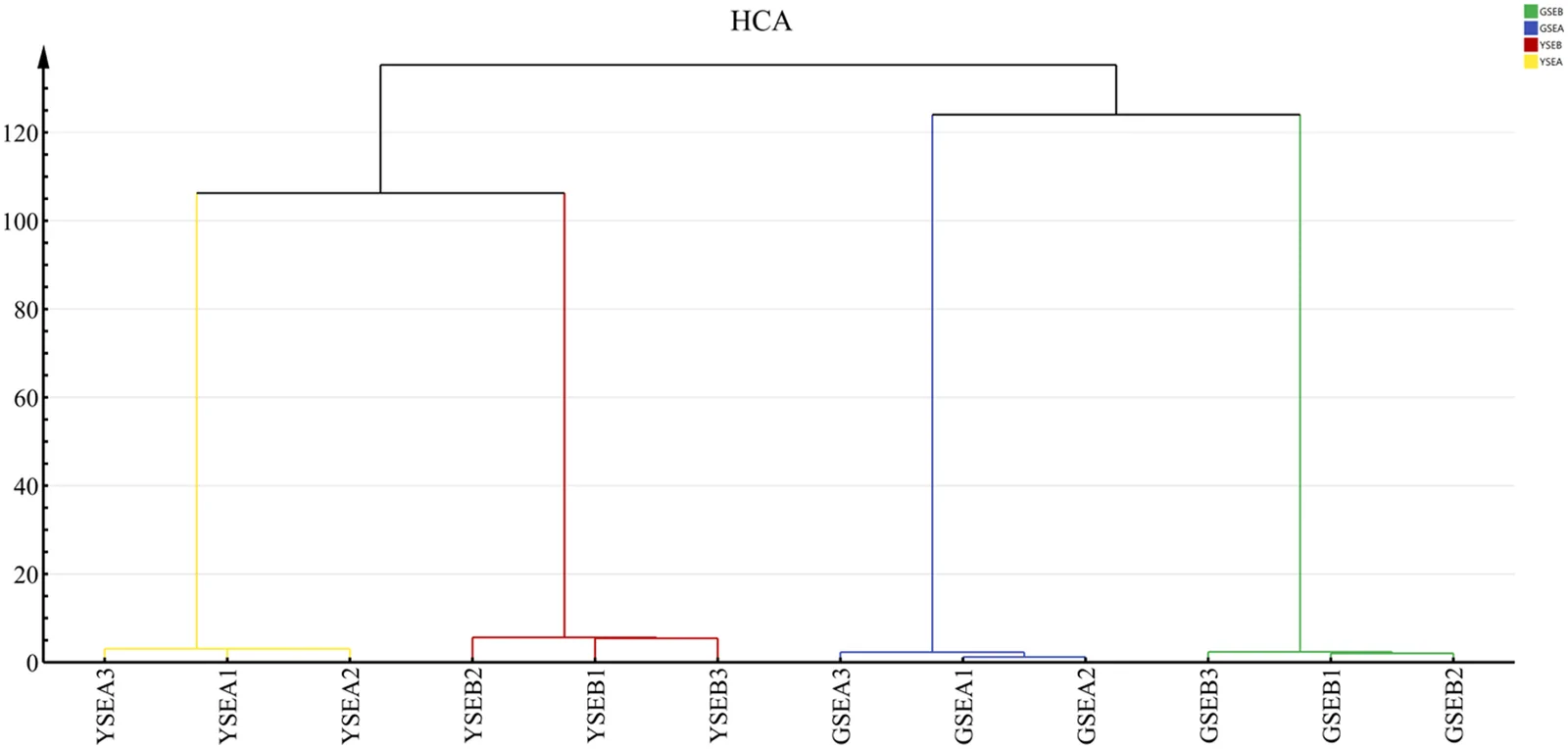
Hierarchical clustering analysis of *Lysimachia foenum-graecum* essential oils.

**Figure 4:**
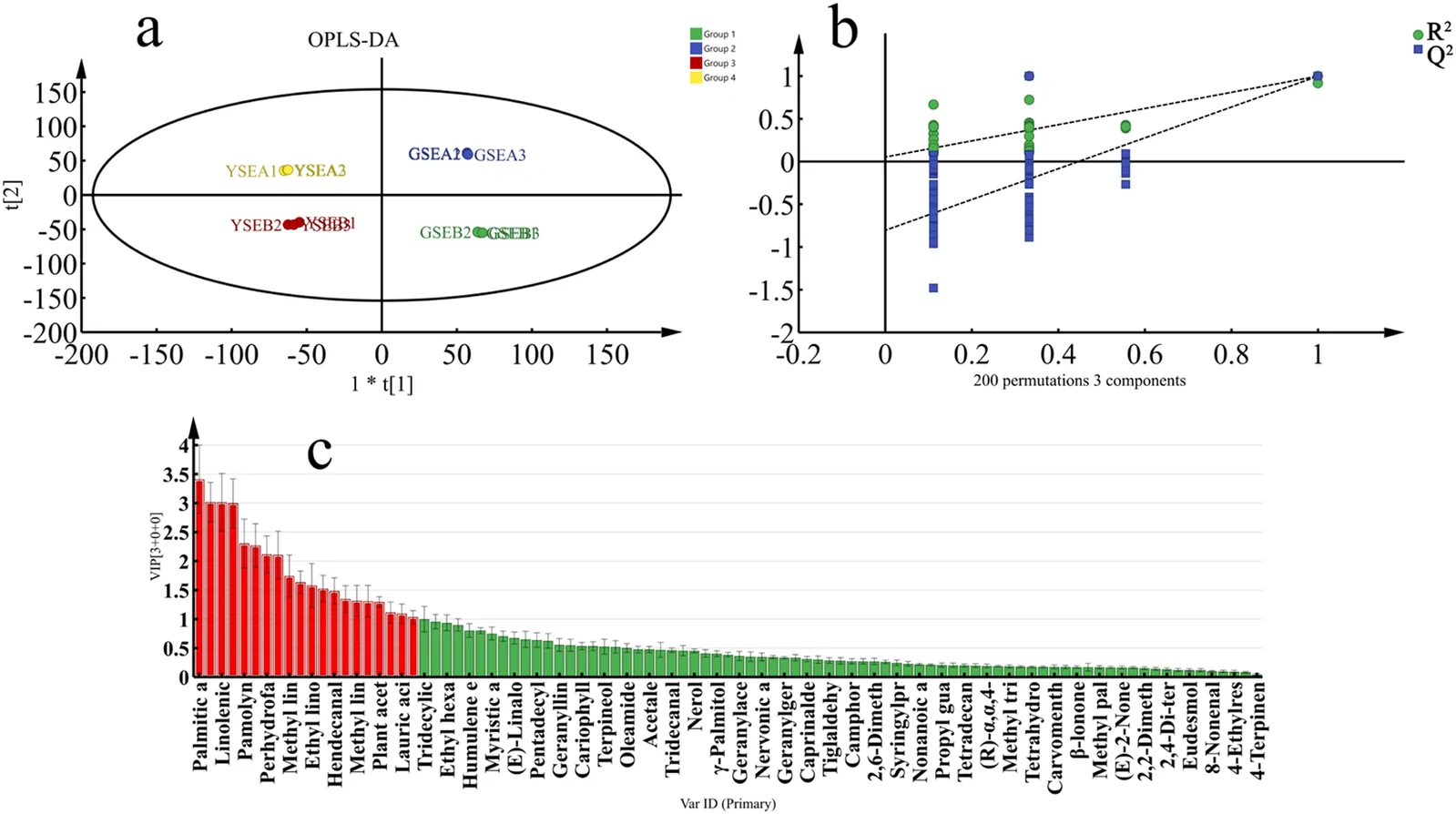
Orthogonal partial least squares-discriminant analysis (OPLS-DA) of *Lysimachia foenum-graecum* essential oils. **(a)** OPLS-DA score plot of the four essential oil samples; **(b)** 200-permutation test of the OPLS-DA model; **(c)** variable importance in projection (VIP) scores of volatile compounds.

The OPLS-DA model exhibited excellent fitting and predictive performance, with R²X, R²Y, and Q² values of 0.997, 0.999, and 0.999, respectively. Since both R² and Q² values were higher than 0.5, the model was considered reliable. Furthermore, 200 permutation tests were performed to evaluate model robustness (**Figure 4b**). The negative intercept of Q² regression line with the Y-axis (−0.745) confirmed that the model was not overfitted and could effectively discriminate different essential oil samples. The contribution of individual volatile compounds to sample discrimination was further evaluated using VIP values (**Figure 4c**). Variables with VIP values greater than 1 were considered important contributors to the classification model. A total of 20 volatile compounds with VIP >1 were identified, including palmitic acid, linoleic acid, linalool, methyl linoleate, pamolyn, etc. The VIP-selected compounds primarily reflected differences in volatile composition among the sample groups and should not be interpreted as definitive key odorants, as VIP values reflect their discriminatory contribution to the multivariate model rather than odor potency or sensory importance. In particular, several long-chain fatty acids and esters have low volatility and may contribute minimally to perceived aroma. Because OAV determination and GC-olfactometry were not performed in the present study, the sensory relevance of individual volatile compounds could not be established based solely on their relative abundances or VIP values. Further investigation combining quantitative analysis with odor thresholds and GC-olfactometry would be required to determine the contribution of individual volatile compounds to specific sensory attributes.

To further determine the chemometric discriminators affected by steam explosion, 20 compounds meeting both VIP >1 and *p* < 0.05 criteria were subjected to hierarchical clustering analysis (**Figure 5**). The clustering results clearly classified the samples into four groups corresponding to untreated Guangxi essential oil, treated Guangxi essential oil, untreated Yunnan essential oil, and treated Yunnan essential oil, confirming that steam explosion induced significant changes in volatile composition. Comparison between untreated and treated samples revealed that the major differential compounds in Guangxi essential oil included cis-9-hexadecenoic acid, methyl linoleate, methyl linolenate, (E)-phytol, ethyl linoleate, ethyl linolenate, and linolenic acid. In Yunnan essential oil, the main differential compounds included linoleic acid, hendecanal, isophytol, and (Z)-11-octadecenoic acid. Most of these differential compounds were long-chain fatty acids and their corresponding esters, which are commonly abundant in plant essential oils. The decreased abundance of these compounds after steam explosion treatment may be associated with the disruption of plant tissues and enhanced migration or degradation during the high-temperature and rapid depressurization process (Bharathi et al., 2024). These results highlight that steam explosion induced selective changes in volatile components, resulting in distinct chemical profiles between untreated and treated essential oils (Ye et al., 2026).

**Figure 5:**
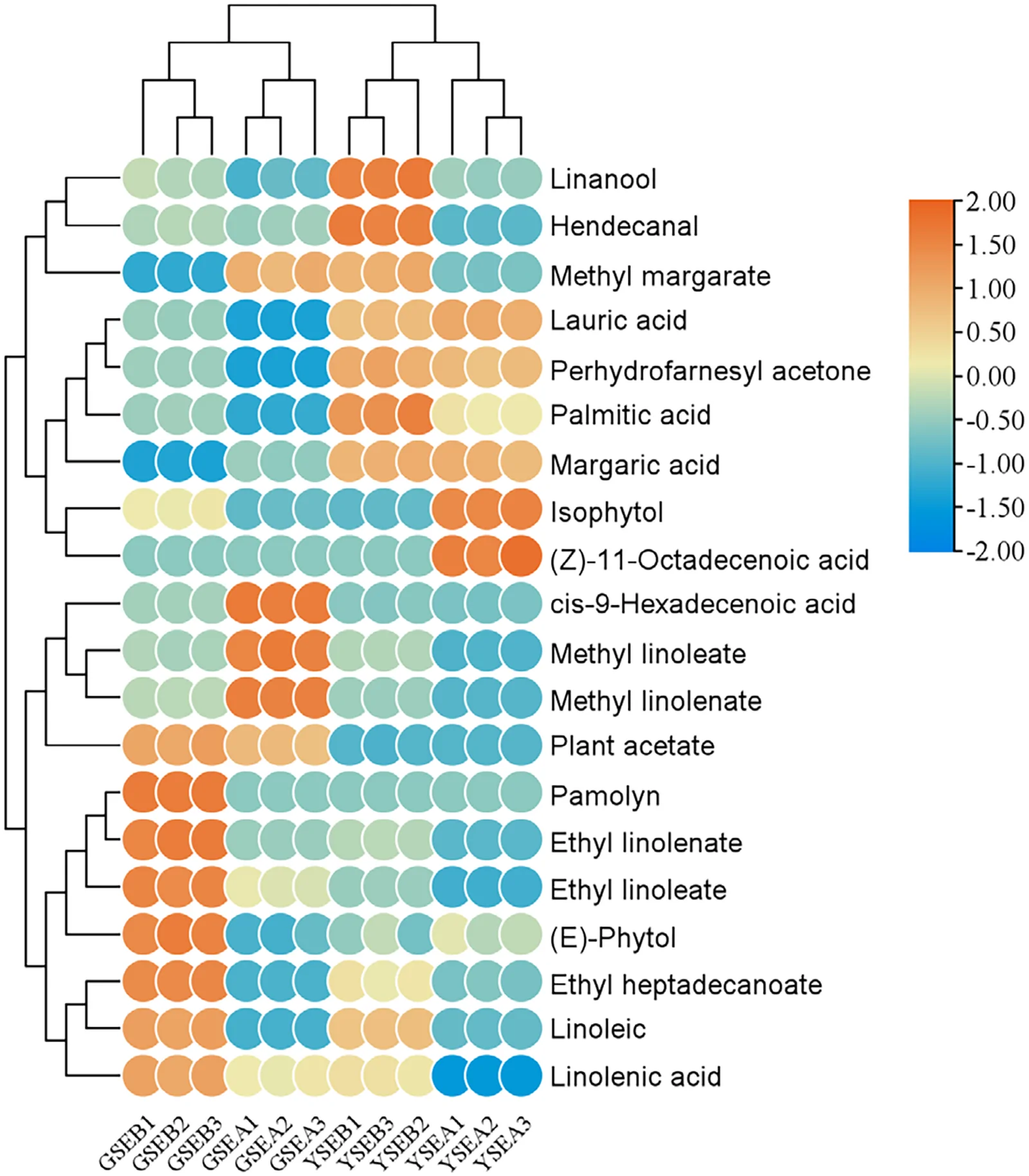
Heatmap analysis of key differential volatile compounds in *Lysimachia foenum-graecum* essential oils.

### 3.5 Sensory characteristics of essential oils before and after steam explosion treatment

The aroma attributes of Guangxi and Yunnan essential oils before and after steam explosion treatment are shown in **Figure 6**. As shown in **Figures 6a, b**, steam explosion treatment altered the overall aroma profiles of both Guangxi and Yunnan essential oils. Compared with untreated samples, the aroma intensities of herbal, floral, sweet, balsamic, woody, and roasted notes generally increased after steam explosion treatment, although the differences were not statistically significant (*p*>0.05, **Supplementary Table S2**). In particular, Guangxi essential oil exhibited a pronounced enhancement in roasted and herbal aroma attributes after treatment, suggesting that steam explosion may promote the formation or release of compounds associated with these aroma characteristics. Combined with the GC-MS results, steam explosion not only reduced the contents of several phenolic compounds and long-chain fatty acids but also induced the formation of newly generated volatile compounds, including megastigmatrienone, furfural, furfuryl alcohol, and 2,5-dimethylpyrazine, which are commonly associated with sweet, roasted, and baked aroma notes. However, the presence of these compounds does not establish their sensory significance without quantitative calibration, odor thresholds, or GC-olfactometry data. The increased roasted scores should therefore be interpreted as a panel-level response to the overall compositional change rather than direct evidence for any single compound. The modification of these volatile components may contribute to the increased aroma complexity of steam-exploded essential oils. The enhanced herbal, sweet, and woody notes after steam explosion align with the aroma profiles previously reported for *L. foenum-graecum* from different origins, where esters, alcohols, and ketones predominated and linalool, borneol, estragole, undecanal, and β-ionone were identified as key aroma-active compounds (Li et al., 2026). Unlike those origin-focused studies, the present work further demonstrates that steam explosion can directionally modulate the aroma profile.

**Figure 6:**
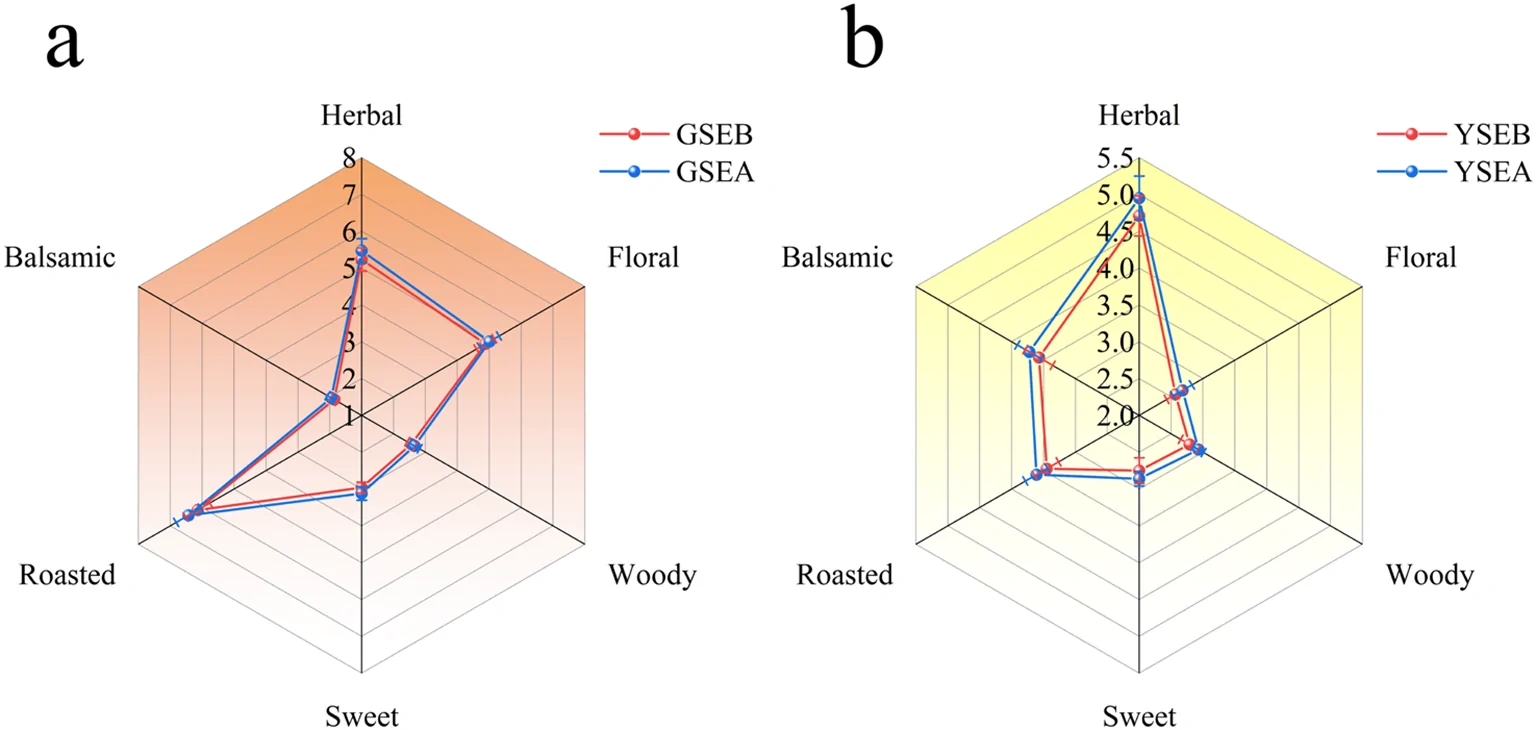
Aroma profiles of *Lysimachia foenum-graecum* essential oils. **(a)** Aroma profiles of Guangxi samples before and after steam explosion treatment; **(b)** aroma profiles of Yunnan samples before and after steam explosion treatment.

## 4 Conclusions

Steam explosion substantially reshaped the GC-MS-detectable chemical profile of *L. foenum-graecum* essential oils, with distinct responses observed between Guangxi and Yunnan materials. The treatment selectively affected different chemical classes, particularly unsaturated fatty acids, their derivatives, and selected terpenoid and phenolic constituents. Chemometric analysis further revealed a distinct separation between untreated and steam-exploded samples, while sensory evaluation showed nonsignificant trends toward increased herbal and roasted attributes following steam explosion treatment. These findings demonstrate that steam explosion alters the volatile composition of L. foenum-graecum and may influence its sensory characteristics, although the observed sensory changes were not statistically significant. However, this modulation is accompanied by a reduction in total GC-MS-detectable volatile constituents, and the causal relationships between individual chemical changes and sensory perception remain to be further established. Therefore, practical application of steam explosion should consider the balance between volatile retention and target aroma characteristics.

## Data Availability

The original contributions presented in the study are included in the article/**Supplementary Material**, further inquiries can be directed to the corresponding author/s.
