# Supplementary material for: Steam explosion-induced remodeling of volatile metabolites in Lysimachia foenum-graecum essential oil revealed by GC-MS and chemometric analysis
Source: Front Plant Sci. 2026 Oct 15;17:1962102. doi: 10.3389/fpls.2026.1962102 (PMC13716804; doi:10.3389/fpls.2026.1962102)
Supplement: Supplementary file 1 [file SupplementaryFile1.docx]

Figure S1 *Lysimachia foenum-graecum* samples before and after steam explosion. (a) *Lysimachia foenum-graecum* sample from Guangxi before steam explosion; (b) *Lysimachia foenum-graecum* sample from Yunnan before steam explosion; (c) the steam explosion machine; (d) *Lysimachia foenum-graecum* sample from Guangxi after steam explosion; (e) *Lysimachia foenum-graecum* sample from Yunnan after steam explosion.

**Table S1** Volatiles detected in *Lysimachia* *foenum-graecum*.

| CAS | Compounds | RI | NIST RI | Matching factor | GSEB (ug/g)^a^ | GSEA (ug/g) | YSEB (ug/g) | YSEA (ug/g) | Level |
| --- | --- | --- | --- | --- | --- | --- | --- | --- | --- |
| 34995-77-2 | (E)-Linalool oxide | 1074 | 1074 | 98 | 24.5±0.44b | 13.78±0.22c | 79.6±0.95a | 6.00±0.13d | L1 |
| 78-70-6 | Linalool | 1104 | 1104 | 100 | 425.99±9.43b | 336.79±12.96d | 681.75±7.86a | 397.44±5.63c | L1 |
| 507-70-0 | Camphor | 1178 | 1177 | 95 | 6.74±0.5c | 5.61±0.06c | 22.08±0.37a | 16.73±1.22b | L1 |
| 562-74-3 | 4-Terpinenol | 1185 | 1184 | 77 | 1.11±0.13b | 1.07±0.04b | 1.57±0.08a | 1.39±0.09a | L1 |
| 98-55-5 | α-Terpineol | 1200 | 1200 | 100 | 160.45±4.33b | 120.55±2.89c | 187.38±8.76a | 150.77±11.36b | L1 |
| 116-26-7 | β-Safranal | 1203 | 1202 | 84 | 6.56±0.02a | 4.76±0.07b | ND | ND | L1 |
| 29548-14-9 | Carvomenthenal | 1223 | 1217 | 85 | 4.01±0.06c | 2.58±0.11d | 9.42±0.38a | 5.64±0.34b | L2 |
| 106-25-2 | Nerol | 1230 | 1230 | 97 | 24.93±0.04b | 21.66±1.56c | 37.26±0.36a | 3.94±0.19d | L1 |
| 106-24-1 | Guaniol | 1256 | 1255 | 98 | 89.65±1.59c | 75.19±3.77d | 129.76±7.54a | 110.86±3.54b | L1 |
| 127-41-3 | Ionone | 1421 | 1427 | 93 | 11.46±0.75d | 13.89±0.28bc | 17.75±0.2a | 14.39±1.98b | L1 |
| 3796-70-1 | Geranylaceton | 1445 | 1452 | 88 | 19.52±0.14b | ND | 27.94±1.75a | 25.83±1.32a | L1 |
| 79-77-6 | β-Ionone | 1478 | 1477 | 87 | 6.34±0.33a | 4.69±0.08b | ND | ND | L1 |
| 18252-44-3 | β-Copaene | 1519 | 1519 | 80 | 7.06±0.15a | 5.07±0.21b | ND | ND | L2 |
| 17092-92-1 | Dihydroactindiolide | 1537 | 1532 | 77 | 4.85±0.08b | 1.67±0.1d | 10.28±0.34a | 3.78±0.08c | L1 |
| 1139-30-6 | Cariophyllene epoxide | 1586 | 1583 | 97 | 67.82±0.18a | 44.78±1.73b | 5.94±0.25c | 4.88±0.31c | L1 |
| 19888-34-7 | Humulene epoxide II | 1612 | 1610 | 97 | 141.1±1.53a | 79.43±1.59b | ND | ND | L2 |
| 502-69-2 | Perhydrofarnesyl acetone | 1841 | 1843 | 96 | 459.93±3.64c | 60.09±1.79d | 1093.86±34.86a | 981.42±24.67b | L1 |
| 1117-52-8 | Farnesyl acetone | 1911 | 1911 | 95 | 118.69±1.78b | 72.95±1.36c | 165.77±1.44a | 53.67±2.67d | L1 |
| 505-32-8 | Isophytol | 1944 | 1943 | 98 | 287.69±4.67b | 162.82±4.79c | 157.75±1.47c | 458.72±4.65a | L1 |
| 1113-21-9 | Geranyllinalol | 2027 | 2034 | 83 | 22.12±1.53b | 67.02±1.62a | 17.07±0.46c | 8.38±0.58d | L1 |
| 150-86-7 | (E)-Phytol | 2114 | 2111 | 99 | 1633.46±14.36a | 1169.51±19.65c | 1260.55±41.86b | 1322.03±24.68b | L1 |
| 24034-73-9 | Geranylgeraniol | 2187 | - | 85 | 31.94±1.46c | 36.03±0.58b | 23.93±0.66d | 43.03±0.37a | L3 |
| 10236-16-5 | Phytyl acetate | 2215 | - | 99 | 425.5±11.54a | 370.3±7.99b | 74.23±4.53c | 78.08±0.77c | L3 |
| 7785-53-7 | (R)-α,α,4-trimethylcyclohex-3-ene-1-methanol | 1201 | - | 82 | ND | 1.98±0.08b | ND | 6.19±0.46a | L3 |
| 38818-55-2 | Megastigmatrienone | 1584 | - | 81 | ND | 3.45±0.12a | ND | ND | L3 |
| 77-53-2 | α-Cedrol | 1611 | 1615 | 95 | ND | 8.89±0.23a | ND | ND | L1 |
| 51317-08-9 | Eudesmol | 1661 | 1655 | 88 | ND | 2.5±0.09a | ND | ND | L1 |
| 1604-34-8 | Tetrahydrogeranylacetone | 1404 | 1407 | 86 | ND | ND | 6.35±0.34a | 5.53±0.31b | L1 |
| 14901-07-6 | Trans-β-Ionone | 1478 | 1486 | 90 | ND | ND | 24.54±0.34a | 20.76±0.79b | L1 |
| 16982-00-6 | Cuparene | 1510 | 1509 | 80 | ND | ND | 14.5±0.11a | 11.21±0.53b | L1 |
| 6750-34-1 | 3,7,11-Trimethyl-1-dodecanol | 1728 | - | 81 | ND | ND | 60.44±1.64a | 42.59±1.64b | L3 |
| **Terpenoids** |  |  |  |  | **3981.42** | **2687.06** | **4109.72** | **3773.26** |  |
| 334-48-5 | Capric acid | 1382 | 1382 | 90 | 27.77±0.13b | 19.86±0.94c | 66.14±2.65a | 23.43±1.37c | L1 |
| 112-37-8 | Undecylic acid | 1475 | 1467 | 92 | 26.27±1.96c | 8.07±0.19d | 80.75±0.28b | 102.85±7.64a | L1 |
| 1883-13-2 | 3-Hydroxydodecanoic acid | 1575 | - | 82 | 14.46±0.96c | ND | 26.03±0.77b | 36.13±2.48a | L3 |
| 143-07-7 | Lauric acid | 1582 | 1580 | 96 | 141.08±1.96c | 40.95±0.98d | 276.81±4.51b | 304.3±4.86a | L1 |
| 638-53-9 | Tridecylic acid | 1677 | 1678 | 93 | 145.56±1.22c | 55.46±1.08d | 228.61±4.54b | 274.65±5.74a | L1 |
| 544-63-8 | Myristic acid | 1775 | 1775 | 96 | 120.92±0.61c | 70.05±1.83d | 201.81±3.16a | 183.7±4.69b | L1 |
| 1002-84-2 | Pentadecylic acid | 1833 | 1833 | 90 | 220.56±1.94c | 165.9±1.54d | 255.44±4.79a | 248.28±0.99b | L1 |
| 3569-10-6 | Valerenic acid | 1881 | 1877 | 79 | 52.27±2.55a | ND | ND | ND | L1 |
| 373-49-9 | cis-9-Hexadecenoic acid | 1950 | 1944 | 98 | 223.52±2.46b | 928.89±8.43a | 150.31±4.05c | 119.43±4.33d | L1 |
| 57-10-3 | Palmitic acid | 1979 | 1979 | 98 | 4250.98±16.77c | 3431.62±17.66d | 6213.07±143.78a | 4907.32±50.63b | L1 |
| 506-37-6 | Nervonic acid | 2012 | - | 86 | 18.26±0.84b | ND | 28.8±1.76a | 19.95±0.12b | L3 |
| 506-12-7 | Margaric acid | 2046 | 2039 | 95 | 2402.75±17.54c | 3132.04±33.55b | 4370.91±43.86a | 4351.61±64.86a | L1 |
| 112-80-1 | Oleic acid | 2047 | - | 82 | 915.71±2.78a | ND | ND | ND | L1 |
| 29743-97-3 | cis-10-Heptadecenoic acid | 2058 | - | 89 | 64.9±1.58a | 27.76±0.75c | ND | 57.8±0.49b | L3 |
| 506-32-1 | Arachidonate | 2066 | - | 84 | 26.87±0.75a | ND | 19.6±0.56b | ND | L3 |
| 60-33-3 | Linoleic acid | 2152 | 2152 | 98 | 1862.96±24.65a | 184.46±1.23d | 1525.37±24.76b | 347.83±5.65c | L1 |
| 463-40-1 | Linolenic acid | 2159 | 2159 | 96 | 2020.12±36.87a | 1299.19±40.76c | 1384.38±33.88b | 15.85±0.17d | L1 |
| 73033-09-7 | cis-10-Nonadecenoic acid | 2230 | - | 77 | 15.67±0.68a | ND | ND | ND | L3 |
| 116-53-0 | Ethylmethylacetic acid | 876 | 878 | 76 | ND | ND | 5.87±0.2a | 4.53±0.09b | L1 |
| 112-05-0 | Nonanoic acid | 1287 | 1283 | 75 | ND | ND | 9.99±0.48a | 8.54±0.23b | L1 |
| 506-17-2 | (Z)-11-Octadecenoic acid | 2152 | - | 91 | ND | ND | ND | 772.92±45.69a | L3 |
| **Fatty acids** |  |  |  |  | **12550.63** | **9364.25** | **14843.89** | **11779.12** |  |
| 2896-60-8 | 4-Ethylresorcinol | 1197 | - | 77 | 1.64±0.13a | 0.15±0.01b | ND | ND | L3 |
| 698-71-5 | 3-Methyl-5-ethylphenol | 1253 | - | 87 | 1.67±0.12a | ND | ND | ND | L3 |
| 2785-89-9 | 4-Ethylguaiacol | 1280 | 1287 | 91 | 16.09±0.66a | 4.76±0.23b | ND | ND | L1 |
| 499-75-2 | Carvacrol | 1323 | 1317 | 78 | 2.45±0.12a | ND | ND | ND | L1 |
| 933-75-5 | 2,3,6-Trichlorophenol | 1367 | - | 87 | 1.18±0.04a | ND | ND | ND | L3 |
| 2785-87-7 | Propyl guaiacol | 1368 | 1371 | 80 | 7.34±0.16a | ND | ND | ND | L1 |
| 14059-92-8 | 2,6-Dimethoxy-4-ethylphenol | 1526 | - | 81 | 15.14±0.47a | 4.86±0.28b | ND | ND | L3 |
| 6766-82-1 | Syringylpropane | 1608 | - | 89 | 12.65±0.47a | 4.27±0.15b | ND | ND | L3 |
| 96-76-4 | 2,4-Di-tert-butylphenol | 1516 | 1518 | 80 | 3.08±0.1a | ND | ND | ND | L1 |
| **Phenols** |  |  |  |  | **61.24** | **14.04** | **0** | **0** |  |
| 105-57-7 | Acetal | 736 | 734 | 87 | 58.29±0.95c | 54.99±2.48c | 104.55±1.86a | 92.6±2.95b | L1 |
| 112-31-2 | Decanal | 1210 | 1207 | 91 | 7.67±0.09c | 5.93±0.14d | 27.25±1.46a | 22.92±0.56b | L1 |
| 112-45-8 | 10-Undecenal | 1304 | 1297 | 97 | 24.14±0.94c | 17.43±0.33d | 214.82±9.54a | 156.82±0.88b | L1 |
| 112-44-7 | Hendecanal | 1311 | 1310 | 99 | 89.36±3.11b | 68.95±3.91c | 352.35±5.78a | 2.66±0.08d | L1 |
| 112-54-9 | Lauraldehyde | 1411 | 1412 | 86 | 7.8±0.98b | 5.52±0.06c | 19.98±0.77a | 1.81±0.07d | L1 |
| 629-80-1 | Palmitaldehyde | 1810 | 1818 | 80 | 14±0.78c | 10.28±0.76d | 36.49±1.65a | 20.23±0.18b | L1 |
| 112-39-0 | Methyl palmitate | 1924 | 1927 | 92 | 77.42±0.86b | 120.73±3.56a | 125.03±3.76a | 20.97±0.57c | L1 |
| 628-97-7 | Ethyl hexadecanoate | 1994 | 1994 | 98 | 210.02±6.54a | 32.01±1.54d | 132.76±0.98b | 76.49±1.24c | L1 |
| 14010-23-2 | Ethyl heptadecanoate | 2061 | - | 96 | 268.55±2.67a | 18.82±0.24d | 141.05±7.43b | 53.59±3.67c | L3 |
| 112-63-0 | Methyl linoleate | 2095 | 2092 | 93 | 100.16±3.67b | 336.65±6.95a | 106.45±0.99b | 21.06±0.57c | L1 |
| 301-00-8 | Methyl linolenate | 2100 | 2101 | 94 | 165.01±1.58b | 584.98±4.37a | 113.57±3.46c | ND | L1 |
| 544-35-4 | Ethyl linoleate | 2164 | 2163 | 97 | 460.25±5.66a | 222.63±8.69b | 144.19±1.86c | 42.76±0.63d | L1 |
| 1191-41-9 | Ethyl linolenate | 2170 | 2169 | 97 | 543.83±13.56a | 98.34±0.76c | 143.03±3.8b | ND | L1 |
| 111-61-5 | Ethyl stearate | 2195 | 2194 | 76 | 35.91±1.76a | ND | ND | ND | L1 |
| 7320-53-8 | 4-Methyldibenzofuran | 1644 | 1639 | 76 | ND | 2.55±0.16a | ND | ND | L1 |
| 1120-25-8 | Methyl palmitoleate | 1904 | 1890 | 79 | ND | 4.77±0.09a | ND | ND | L1 |
| 1731-92-6 | Methyl margarate | 1989 | 2001 | 95 | ND | 121.76±4.59a | 123.3±4.54a | 29.68±0.66b | L1 |
| 112-61-8 | Methyl octadecanoate | 2130 | 2128 | 86 | ND | 12.43±0.12a | ND | ND | L1 |
| 497-03-0 | Tiglaldehyde | 755 | 749 | 80 | ND | ND | 16.82±0.49a | 8.88±0.36b | L1 |
| 39770-04-2 | 8-Nonenal | 1102 | - | 80 | ND | ND | 2.12±0.08a | 1.89±0.11b | L3 |
| 41519-23-7 | (Z)-3-Hexenyl isobutanoate | 1109 | - | 85 | ND | ND | 47.8±0.88a | 33.65±0.73b | L1 |
| 3809-32-3 | 2,5-Dimethylcyclohexanol | 1114 | 1099 | 80 | ND | ND | 7.06±0.45a | 6.76±0.09a | L1 |
| 18829-56-6 | (E)-2-Nonenal | 1167 | 1162 | 83 | ND | ND | 5.84±0.08a | 3.69±0.12b | L1 |
| 112-30-1 | 1-Decanol | 1278 | 1278 | 85 | ND | ND | 6.37±0.5a | 4.33±0.35b | L1 |
| 112-38-9 | Undecenoic acid | 1469 | - | 83 | ND | ND | 8.23±0.17a | 4.55±0.28b | L3 |
| 10486-19-8 | Tridecanaldehyde | 1515 | 1503 | 92 | ND | ND | 43.89±0.17a | 39.23±2.68b | L1 |
| 5129-56-6 | Methyl 10-methylundecanoate | 1528 | - | 77 | ND | ND | 5.62±0.09a | 4.31±0.28b | L3 |
| 124-25-4 | Tetradecanal | 1618 | 1618 | 88 | ND | ND | 8.47±0.38a | 5.63±0.36b | L1 |
| 1731-88-0 | Methyl tridecanoate | 1629 | 1631 | 75 | ND | ND | 7.21±0.15a | 4.69±0.18b | L1 |
| 2345-28-0 | 2-Pentadecanone | 1700 | 1698 | 78 | ND | ND | 8.9±0.16a | 8.61±0.57a | L1 |
| 2765-11-9 | 1-Pentadecanal | 1719 | 1715 | 97 | ND | ND | 74.12±0.17a | 24.68±0.76b | L1 |
| 301-02-0 | Oleamide | 2368 | 2375 | 90 | ND | ND | 53.49±2.65a | 39.15±0.81b | L2 |
| 2370-14-1 | 2,2-Dimethyloctanol | 1099 | - | 75 | ND | ND | ND | 3.5±0.17a | L3 |
| 730-46-1 | γ-Palmitolactone | 2109 | 2106 | 77 | ND | ND | ND | 24.3±0.2a | L1 |
| **Others** |  |  |  |  | **2062.41** | **1718.77** | **2080.76** | **759.44** |  |

^a^ “ND”, Not detected; Different lowercase letters within the same row indicate significant differences among the four samples according to one-way ANOVA followed by Duncun's test (*p* < 0.05).

Table S2 Significance analysis of the sensory evaluation.

| Attributes | Significance^a^ | |
| --- | --- | --- |
|  | GSEB vs GSEA | YSEB vs YSEA |
| Herbal | 0.496 | 0.448 |
| Floral | 0.677 | 0.485 |
| Woody | 0.524 | 0.282 |
| Sweet | 0.397 | 0.480 |
| Roasted | 0.491 | 0.424 |
| Balsamic | 0.459 | 0.500 |

^a^ Values represent p-values obtained from two-sample Student's t-tests comparing sensory attributes before and after steam explosion treatment. All p-values were greater than 0.05, indicating no statistically significant differences..
